# The association of cognitive representations with psychological adjustment in experience of infertility and fertility treatment: A systematic review

**DOI:** 10.18332/ejm/138598

**Published:** 2021-08-02

**Authors:** Meropi Moutzouri, Antigoni Sarantaki, Kleanthi Gourounti

**Affiliations:** 1 Department of Midwifery, School of Health and Care Sciences, University of West Attica, Athens, Greece

**Keywords:** infertility, IVF, cognitive representation, anxiety, depression, well-being

## Abstract

**INTRODUCTION:**

The aim of this systematic review was to examine studies describing the association of cognitive representations with psychological adjustment or maladjustment during the experience of infertility and its treatment in light of the Common-Sense Model. According to this theoretical model cognitive perceptions about an illness can be associated with emotional adaptation.

**METHODS:**

A systematic search of four electronic databases (PubMed, APA PsycINFO, SCOPUS, ScienceDirect) was performed. This review considered only quantitative, primary studies in the English language without geographical limitations, published during the period 1996–2020 and relevant to the objective. The population of interest was infertile individuals who are having or not having infertility treatment. Only studies that examined the association between independent variables, such as perceived causes, timeline, controllability, consequences, symptoms, illness coherence and emotional representations, with psychological variables, such as anxiety, worry, distress, depression and well-being, were included. Two authors performed an independent extraction of articles using predefined data fields. Relevant articles were critically appraised and a narrative synthesis was conducted.

**RESULTS:**

Seven cross-sectional studies met the inclusion and methodological criteria and were included in the review. The review results revealed that all components of cognitive representations of infertility and its treatment may correlate with psychological adaptation of people who deal with a fertility problem, at intrapersonal and interpersonal level.

**CONCLUSIONS:**

This systematic review suggested that the Common-Sense Model is an appropriate theoretical model to be applied in the experience of infertility and health professionals can make interventions based on modifying cognitive perceptions of a fertility problem that may increase levels of psychological well-being and decrease levels of distress.

## INTRODUCTION

Psychological adjustment during infertility and fertility treatment and factors that are correlated with this adjustment, have to be examined, in order that infertile people who are vulnerable to higher distress are detected early by health professionals and offered effective psychological intervention. These factors may comprise cognitive representations (perceptions, beliefs) about the experience of infertility.

According to Common-Sense Model (CSM) that is based on self-regulation theory^1^, patients form cognitive representations regarding causes, timeline, controllability, consequences and symptoms of health threat so as to understand and cope with it. Moss-Morris et al.^2^ added two more aspects of cognitive representations, namely illness coherence, which refers to clear picture and understanding of a health problem, and emotional representations, which refers to how emotional patients felt in relation to their problem. The way that patients perceive a health problem ultimately affects the coping strategies and the emotional adaptation to it.

CSM is one of the most developed and most research-tested theoretical models concerning the beliefs related to health threats^3^. This model has been applied for assessing the relationship between beliefs and emotional adaptation to various chronic medical diseases such as psoriasis^4,5^, rheumatoid arthritis^5,6^, myocardial infarction^7^ or cardiovascular problems^8^, chronic pain^9^ and in various types of cancers^10-12^. According to a meta-analysis, there is strong evidence that several illness perceptions can predict psychological adjustment to patients of various chronic diseases in light of CSM^13^.

Despite that CSM has been designed for patients of chronic illnesses, it may prove a useful theoretical model in studying adjustment of infertile couples, as infertility has common features with chronic diseases. Regarding their similarities, the fertility problem often arises from anatomical and organic dysfunctions of the reproductive system of either male or female, or both. Usually both patients of chronic diseases and infertile persons experience the health issue for a long period of time^14^. Furthermore, infertile couples usually appraise infertility as a state of low control as they think that no or little action can be taken to deal with it, an appraisal that patients of chronic diseases form as well^14^. Concerning the consequences, the experience of infertility may have a negative impact on various aspects of life, such as a social impact or an impact on marital relationship, impacts that patients with chronic illnesses might experience also^15^. All of these perceptions regarding timeline, controllability and consequences of experience of infertility support, allow infertile persons to undergo a self-regulation process and to try and regulate themselves to a state of normality, such as fertility.

The aim of this review was to investigate the association between cognitive representations and psychological adjustment during the experience of infertility and during fertility treatment.

## METHODS

### Eligibility criteria

There are two general categories of eligibility criteria: study characteristics and report characteristics^16,17^. In this systematic review, studies were selected according to the criteria outlined below.

#### Study eligibility criteria

Quantitative surveys were included, whereas reviews and qualitative studies were excluded. Articles had to be based on participants of any age, women or men, experiencing infertility or its treatment (e.g. via IVF). Each study had to be conducted in the light of CSM. Articles had to examine the association between cognitive representations and psychological outcomes, using scales of illness perceptions so as to measure cognitive representations. Articles had to use questionnaires of negative or positive psychological adjustment as outcome variables, such as depression, anxiety, quality of life, and well-being.

#### Review eligibility criteria

In this systematic review only articles in English were accepted due to lack of translation resources. There were no geographical limitations. There was a limitation with regard to year of the research. The articles had to be published between 1996 and 2020, since the Illness Perception Questionnaire (IPQ), the first tool of measuring illness perception according to CSM, was published by Weinman et al.^18^ that year.

### Literature search strategy

A systematic search of four medical and psychological electronic bibliographic databases was conducted at the middle of 2020, through PubMed, APA PsycINFO, SCOPUS and ScienceDirect. The search of literature via electronic databases was initiated from 1996 until 2020. The literature search was limited to the English language. The specific search strategies were created by author KG who had a previous experience in systematic review searching. Two authors developed and carried out the search.

A robust search strategy was developed using appropriate Medical Subject Headings (MeSH terms) and associated text words. As many synonyms as possible were included to ensure that all potentially useful articles were included. Search terms, such as Common-Sense Model, self-regulation model, and Leventhal’s model were used to find surveys that relied on CSM as the theoretical framework. Also, various search terms were used to cover the concept of perceptions in the experience of infertility, such as illness perception, illness representation, cognitive beliefs, cognitive appraisal, perception, belief, and controllability. Moreover, different search terms were used in order to define the concept of infertility, such as infertility, infertile, *in vitro* fertilization, and IVF. The three concepts and their associated text words were combined using Boolean phrases, using AND and OR where necessary. Techniques such as truncation, denoted by an asterisk, and enclosed quotation marks, were used when required; the former to search for various spellings and the latter to ensure words appeared together. The search of key concepts and their synonyms was conducted on titles, abstracts, keywords, and texts. Furthermore, other methods of searching were used, such as searching the reference sections of selected articles to retrieve additional records that might not have been picked up by the electronic search and manual searching of relevant journals. Finally, the systematic review team circulated the bibliography of the included articles.

### Study selection

All references retrieved during the systematic research were stored in Mendeley. Two review authors independently screened the bibliography. Titles and abstracts were screened for eligibility criteria. Articles, whose abstracts alluded to the search topic or where there was any uncertainty, were selected for full-text screening. Review author pairs then decided whether these articles met the inclusion criteria and if relevant, data were extracted and recorded for inclusion in this review. A systematic review data management software was not used due to the limited number of relevant reports. The level of inter-rated agreement between the two authors was high.

### Data extraction

Using a pilot data extraction form, that had been developed a priori, one review author extracted the data from included studies and then the second author checked the extracted data. Disagreements were resolved by discussion between the two review authors. A systematic review data extraction software was not used due to the limited number of relevant reports. Information was extracted from each included study on participants, cognitive perceptions, and psychological adjustment. A summary of each publication included in this review (Table 1) and the key findings of each study (Table 2) were extracted and recorded in preparation for data synthesis.

**Table 1:** Characteristics of included studies

*Authors, year*	*Country*	*Study design, assessment time*	*Sample size*	*Standardized measures of cognitive perceptions*	*Standardized measures of emotional adjustment*
Benyamini et al.^23^ 2004	Israel	Cross-sectional, during various treatments of infertility	310 women	IPQ (timeline, consequences, controllability)	Infertility Specific Well-being and Distress Scale
Benyamini et al.^22^ 2009	Israel	Cross-sectional, first visit to an infertility clinic or at various stages of treatment	Sample 1: 72 couples, Sample 2: 49 couples	IPQ (timeline, consequences, controllability)	Infertility Specific Well-being and Distress Scale
Benyamini et al.^21^ 2016	Israel	Cross-sectional, during infertility treatment	194 women	IPQ-R (timeline, consequences, controllability, coherence, 3 types of controllability)	Infertility Specific Well-being and Distress Scale
Gourounti et al.^25^ 2012	Greece	Cross-sectional, during IVF	137 women	IPQ-R (Personal and treatment controllability)	(STAI)-State, CES-D
Grinberg^27^ 2016	Finland	Cross-sectional, during IVF	80 women	IPQ-R (all subscales)	Multidimensional Quality of Life Questionnaire
Lord and Robertson^24^ 2005	United Kingdom	Cross-sectional, period before ICSI or frozen embryo transfer	30 women, 20 men	IPQ-R (all subscales)	HADS
Naab et al.^26^ 2013	Ghana	Cross-sectional, during infertility treatment	203 women	FBQ (timeline, consequence, illness coherence, personal control, treatment control)	BAI, CES-D

**Table 2:** Studies examining the association of aspects of cognitive representations with psychological adjustment during the experience of infertility and its treatment

*Authors, year*	*Association of aspects of cognitive perceptions with psychological adjustment – intrapersonal level*
Benyamini et al.^23^ 2004	Timeline, consequences, controllability
Benyamini et al.^22^ 2009	For men: timeline, consequences, controllability For women: controllability consequences
Benyamini et al.^21^ 2016	Consequences, control over the treatment procedure
Gourounti et al.^25^ 2012	Personal control
Grinberg^27^ 2016	All cognitive perceptions except of controllability
Lord and Robertson^24^ 2005	Causes, timeline
Naab et al.^26^ 2013	Coherence, consequences, controllability

### Quality assessment of the reviewed articles

The seven studies were evaluated for methodological quality, using a structured format adapted from Greenhalgh^19^. The systematic review as conducted adhered to the PRISMA (Preferred Reporting Items for Systematic Reviews and Meta-Analysis) guidelines^20^. Criteria for evaluating methodological quality of included studies were: 1) Evidence of random recruitment of participants; 2) response rate ≥70%; 3) evidence that each variable, such as cognitive perceptions, distress, anxiety, depression, and well-being, was measured by using standardized, validated instruments; and 4) control of confounding variables for avoiding systematic bias. Studies that provided evidence of measuring each variable by using standardized, validated instruments (3rd criterion) and met at least two out of the remaining three methodological quality criteria were finally included. Quality assessment and data extraction was conducted by a single reviewer, using these explicit criteria. A second reviewer then checked the procedure of quality assessment.

### Data synthesis

A systematic narrative synthesis was provided with information presented in the text and in Tables 1 and 2, so as to summarize and explain the characteristics and the key findings of each study. The narrative synthesis explored the relationship and findings both within and between included studies.

## RESULTS

### Study selection

**Figure 1:**
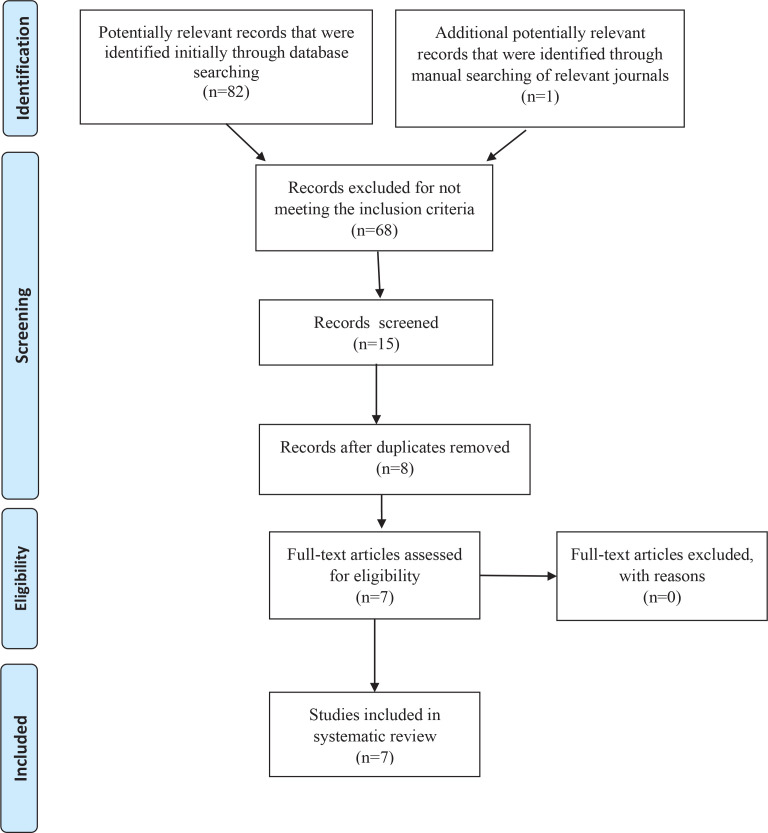
Selection process of included studies

The initial search generated 83 titles. After the assessment of the titles, abstracts and full-texts, 68 references were excluded because they were not relevant to the search topic and they did not fit the selection criteria of this review. Fifteen articles were selected, 8 of which were duplicates, leaving 7 studies for this systematic review. The process for identification, screening, eligibility and inclusion, which underpins this systematic search, is illustrated by the flowchart depicted in Figure 1. The seven studies included in this systematic review are described in Table 1.

### Included studies characteristics

#### Study location

Three of the seven studies took place in Israel^21-23^, one study in the UK^24^, one survey in Greece^25^, one study in Ghana^26^ and one study in Finland^27^.

#### Study design

All of included studies were cross-sectional. Included studies used specific inclusion criteria for controlling certain confounding demographic (e.g. age) and medical (e.g. duration of infertility, fertility treatments received) variables in order to address potential sources of bias. In addition, included studies incorporated statistical analyses, such as hierarchical linear or logistic regression, for controlling confounding variables.

#### Participants

Samples of all accepted studies consisted of infertile individuals, but participants did not experience the same stage of infertility or its treatment. In the survey of Benyamini et al.^23^, infertile individuals were receiving ovulation-inducing medication in the form of pills or by injection, or were undergoing IVF, or were experiencing other treatments, or had not yet begun any treatment. The study included both couples who experienced their first visit to a specialist infertility clinic and couples who had a regular visit to a specialist infertility clinic; participants were undergoing infertility treatment. In study of Gourounti et al.^25^ participants were undergoing an IVF cycle. In a study of Grinberg^27^ the sample was approached during a visit to the IVF unit. In the study of Lord and Lobertson^24^ participants were attending a clinic to discuss and/or plan a cycle of *in vitro* fertilization (IVF). Naab et al.^26^ included at their survey infertile participants who were receiving treatment for fertility problems.

The size of survey samples ranged from 50 to 310 infertile participants. Five studies included more than 100 participants^21-23,25,26^, while two studies included less than 100 participants^24,27^. Six studies did not have a comparison group^21,23-27^. One of the screened studies included two matched control groups of which one sample consisted of couples who were experiencing the first time they were referred to a clinic that specializes in infertility, and a second sample included couples at a regular visit to a specialist infertility clinic^22^. Both infertile men and women were participants in two studies^22,24^, while five studies included only infertile women^21,23,25-27^. Only one study in this review provided a power calculation a *priori* ^25^, while another study performed a *post hoc* power analysis, which indicated that the effect size was large and the power of the tests was good^24^.

### Instruments of cognitive representations

Timeline, consequences and controllability subscales of Illness Perception Questionnaire (IPQ) were administered in two researches^22,23^. The Illness Perception Questionnaire Revised (IPQ-R)2 was distributed to the participants in four studies^21,24,25,27^, but different subscales of this questionnaire were used by each study. In particular, in study of Benyamini^21^ were used subscales of timeline, consequences, illness coherence and of three types of controllability (self-control over fertility problem, treatment control, self-control over treatment procedure). In the study of Gourounti et al.^25^ personal and treatment control subscales were used, and in the surveys of Lord and Robertson^24^ and Grinberg^27^, all the subscales of IPQ-R (identity, timeline, consequences, self-control of the problem and the treatment, problem coherence, emotional representations and causal subscales) were administered to infertile participants. In the research of Naab et al.^26^, subscales of consequences, illness coherence and personal control of the Fertility Beliefs Questionnaire (FBQ) were given to infertile couples.

### Instruments of psychological adjustment

Psychological outcomes were conceptualized in terms of self-reported symptoms of anxiety, worry, distress, depression, and well-being. Psychological variables were assessed by general and infertility-specific validated tools. Emotional maladjustment was assessed by three studies using different measurement tools. In the study of Gourounti et al.^25^, the state of anxiety was measured by the State Trait Anxiety Inventory, and the Centre for Epidemiologic Studies-Depression (CES-D) was used to assess depressive mood of participants. The Hospital Anxiety and Depression Scale was used by Lord and Robertson^24^. In the survey of Naab et al.^26^, anxiety was measured by Beck’s Anxiety Inventory (BAI), and the Centre for Epidemiologic Studies-Depression (CES-D) was used to assess depressive symptoms of infertile individuals. Moreover, emotional maladjustment and adjustment were assessed in three surveys also. The short version of the Infertility-Specific Well-being and Distress Scales was administered to the participants of three studies^21-23^. Furthermore, quality of life, as a variable of positive adjustment, was measured by the Multidimensional Quality of Life Questionnaire in the study of Grinberg^27^.

### Outcomes: The association of cognitive representations with psychological adjustment

The key findings of each study concerning the association of aspects of cognitive representations with psychological adaptation, at intrapersonal and interpersonal level, are presented in Table 2. Six studies investigated the association of cognitive perceptions with psychological adaptation of infertile individuals at intrapersonal level. Benyamini et al.^23^ suggested that infertile women’s perceptions of long period fertility problem, of serious effects, and of reduced control over a fertility problem, were positively associated with increased levels of psychological distress and lower well-being. They noticed that the aspect of consequences had the strongest association with the adjustment outcomes of infertile women. Also, Lord and Robertson^24^ found that stress as a perceived cause of infertility and perceived cyclical timeline was associated with increased levels of anxiety and depression, but they pointed out that the majority of participants did not show clinical signs of psychopathology, such as depression. Moreover, Gourounti et al.^25^ showed that perception of reduced personal control is associated with increased levels of fertility related stress and state anxiety, but not with depression in women with a fertility problem. Furthermore, Naab et al.^26^ concluded that beliefs that infertility had negative consequences and poor illness coherence, were related to increased infertility related stress, anxiety and depressive symptoms. They pointed out also that perception that infertility could be managed by personal control was significantly correlated with lower levels of anxiety. Also, Benyamini et al.^21^ concluded that perceived consequences were strongly related to distress and well-being of infertile women. They also examined the role of three types of controllability, self-control over the problem, treatment control, and self-control over the treatment procedures, on well-being and psychological distress of women undergoing infertility treatments. They concluded that the greater the personal control over medical procedures, the greater the levels of well-being and the lower the levels of psychological distress experienced by women. Grinberg^27^ concluded that the more negative a woman’s perceptions about symptoms, timeline, consequences, emotional representation, problem coherence and causal attributions, the lower the quality of life she experienced.

Only one study investigated the relation of cognitive perceptions with psychological adaptation of infertile individuals at intrapersonal and interpersonal level too. Benyamini et al.^22^ examined the cognitive perceptions of infertile men and women, and their associations with psychological adjustment through dyadic analyses. At intrapersonal level, they concluded that cognitive representations of consequences, timeline, and controllability, were associated with emotional adaptation in men, while only cognitive representations of consequences were associated with women’s psychological outcomes. At interpersonal level, they found out that person’s psychological distress was associated with both his/her belief about serious consequences of infertility and his/ her partner’s similar belief. Moreover, they concluded that incongruence of two spouses in cognitive representations was correlated with psychological distress, especially in women. For example, women who believed that they had limited control over the experience of infertility had higher levels of psychological distress when their husbands had more positive beliefs about controllability, compared to infertile women who perceived control as negatively as their partners.

## DISCUSSION

The aim of this review was to investigate the association of cognitive representations with psychological adjustment of infertile people. The finding of this systematic review is that all components of cognitive representations (causes, timeline, consequences, controllability, identity, illness coherence) of infertility and its treatment may be correlated with psychological adaptation of people who deal with a fertility problem. In general terms, the more negative the person’s representations of infertility and its treatment, the more negative his/her emotional response.

### Causes

With regard to the positive association of causal dimension of cognitive representations with the decreased levels of quality of life and increased levels of anxiety and depression^24,27^, a possible explanation of these findings is that women who perceive stress as the cause of their experience of involuntary childlessness, are less likely to have a clear understanding of their problem, which the CSM suggests is important for psychological well adaptation. Another explanation might be, that women, who believe that stress caused their fertility problem, essentially blame themselves for having stress and for not being able to control it, which leads to increased emotional maladjustment.

### Timeline

Regarding the positive relation of longer or cyclical timeline of experience of infertility and its treatment with decreased quality of life and increased distress in infertile men or women^22-24,27^, an explanation may be that perception of chronic or cyclical duration of infertility refers to the belief of absence of a quick and immediate solution of a fertility problem, belief that could exhaust psychologically infertile individuals.

### Consequences

With regard to the association of representations of consequences with high levels of distress and low levels of well-being of infertile people^21-23,26,27^, a possible explanation of the association of perceived effects and psychological maladjustment is that infertile women, whose bodies undergo most of the medical examinations, procedures and treatments, believe that the fertility problem has major consequences on various domains of their daily life, which leads to suffering from anxiety, depression, and decreased well-being. Also, this might happen due to the fact that some studies were conducted in a religious community where societal pressures for childbearing are very strong and where women may feel these pressures more strongly, a situation that leads to higher levels of distress in infertile women.

### Controllability

According to representations of controllability, results of included studies concluded that infertility and its treatment seem to be low control situations^21-23,25^. When controllability over the fertility problem or the treatment outcomes is not possible, people may deal with psychological maladjustment.

### Identity

Regarding the cognitive representations of identity, the more physical symptoms an infertile woman suffers from, such as pain, weight loss/gain, fatigue, and redness at her body parts due to injections, the lower is her level of well-being^27^. An explanation of this is the fact that experiencing bodily symptoms contributes to perceptions of infertility and its medical treatment as a health problem or threat, and consequently results in a decreased quality of life of infertile individuals.

### Illness coherence

Moreover, regarding the relation of illness coherence with increased distress and decreased well-being^26,27^, an explanation might be the fact that infertility is characterized by vagueness and uncertainty of ability to conceive and its treatment is characterized by rates of unsuccessfulness and difficulty to understand the complicated medical information and procedures, facts that lead to an absence of clarity, an increase of worry, stress and anxiety and a decrease of well-being levels. Leventhal et al.^1^ suggested that it is only once people have interpreted or have made sense of their problem that they can begin to try, cope with and solve it, until then the psychological reaction to that problem is probably to be characterized by worry and maladjustment.

In summary, all components of cognitive representations of infertility and its treatment may be associated with psychological positive adaptation or maladjustment of people who deal with fertility problem, at intrapersonal and interpersonal level. The data underscored the importance of examining the self-regulation process during the experience of infertility and its treatment.

However, the seven included studies did not show coherence in terms of their methodology. These differences make comparisons a difficult procedure. Regarding their methodology, researchers did not use the same methodological tool for estimating cognitive representations or the same questionnaire in order to measure emotional outcomes. Also, some studies used only measurements of psychological maladjustment, such as stress, anxiety, depression and distress, while others used instruments of negative and positive adjustment too or used only tools of positive adaptation. Some methodological differences that refer to samples are, that participants of included studies did not experience the same stage of infertility or its treatment in all of surveys or that all studies did not include both men and women of the infertile couple.

### Limitations

The findings of this review should be interpreted after considering some methodological limitations. First, this review included only quantitative studies and articles in English. Studies that used a qualitative or mixed methodology design were excluded and, thus, a deeper understanding of the effect of perceptions on psychological outcomes might be restricted to some extent. In addition, studies that were written in other than the English language were excluded, and this may have limited the findings of this systematic review. Furthermore, the failure of some published studies to emerge during the electronic search might have affected the present review. However, the dissemination bias was diminished by examining the reference list of retrieved articles and searching the reference list of previous review articles.

## CONCLUSIONS

### Implications for practice

According to included studies in this systematic review, cognitive representations are associated with psychological adjustment of individuals experiencing involuntary childlessness. CSM is an appropriate theoretical framework to understand and approach the experience of infertility and its treatment. The next step is to translate this knowledge into effective interventions. Health professionals can detect the presence of risk cognitive factors in infertile couples during first visits in a fertility clinic and make interventions based on modifying individuals’ perceptions in order to reduce their distress and increase their well-being.

### Implications for research

A number of scientific steps have to be made. Research that refers to relation of perceptions with emotional adjustment in experience of infertility and its treatment is too limited. Seven surveys have been published in the last 16 years examining the role of cognitive representations on emotional adaptation of infertile individual, at intrapersonal or interpersonal level, in the light of CSM. More relevant research needs to be conducted.

Moreover, the majority of the literature is based on examining the association of beliefs with mental health at intrapersonal level. More research needs to be conducted in order to examine the association of perceptions with psychological health at interpersonal level too, examining the dyadic, dissimilarity and interaction effects of cognitive representations on psychological outcome of both members of infertile couples, because of the dyadic and shared nature of the experience of fertility problem and its treatment.

Another issue refers to the use of matched control groups and the representativeness of samples. The majority of the included studies did not use a control group, an issue often reported as a methodological weakness. Health professionals and researchers meet infertile people who are most to seek help. Due to this, findings are more likely to be correlated with the beliefs of people undergoing a treatment for a fertility problem rather than the cognitive perceptions of the infertile individuals as a whole.

Furthermore, research of cognitive perceptions can benefit from the usage of all subscales of the same questionnaire of cognitive representations. Also, study on emotional adjustment can benefit from the usage of more infertility specific and more homogenous scales of psychological outcomes. Moreover, outcomes of positive adjustment, such as well-being, except of maladjustment, have to be examined.

Also, included studies assessed emotional adjustment at different time periods of experiencing infertility or at various stages of its treatment, as experience of fertility problem and its treatment is a long process. Research needs to be conducted at a specific time and distinct stages of experience of involuntary childlessness and its treatment to compare their results and examine the effect of time/stage on the association between beliefs and psychological adaptation.

In addition, future studies should assess distress and well-being after controlling for different confounding variables, such as demographic variables, previous history of infertility treatments, type of treatment or method of assisted reproduction, and stage of treatment. For example, cultural, religious and social variables may influence perceptions of infertility and consequently psychological outcomes of infertile people. Also, information from medical history, such as number of unsuccessful IVFs in the past, may contribute to the associations between perceptions and mental health.

## Data Availability

Data sharing is not applicable to this article as no new data were created.
